# Effect of vitamin D_3_ on the antimicrobial activity of human airway surface liquid: preliminary results of a randomised placebo-controlled double-blind trial

**DOI:** 10.1136/bmjresp-2017-000211

**Published:** 2017-06-04

**Authors:** Luis G Vargas Buonfiglio, Marlene Cano, Alejandro A Pezzulo, Oriana G Vanegas Calderon, Joseph Zabner, Alicia K Gerke, Alejandro P Comellas

**Affiliations:** 1 Department of Internal Medicine, Roy J and Lucille A Carver College of Medicine, University of Iowa, Iowa City, Iowa, USA; 2 Department of Pediatrics, Roy J and Lucille A Carver College of Medicine, University of Iowa, Iowa City, Iowa, USA

**Keywords:** Airway Epithelium, Innate Immunity, Bacterial Infection, Respiratory Infection, Tobacco and the lung

## Abstract

**Introduction:**

Vitamin D_3_ supplementation has been reported to prevent lung infections and increase the gene expression of antimicrobial peptides such as cathelicidin. We investigated the effect of vitamin D_3_ supplementation on the antimicrobial activity of airway surface liquid (ASL) in human subjects. Since smoking can increase the risk of respiratory infections, we also investigated the effect of smoking in the cathelicidin response to vitamin D_3_ in human airway epithelia in vitro.

**Methods:**

This study is a subanalysis of single-centre community-based randomised placebo-controlled double-blind trial. Participants were randomised to receive 1000 international units per day of oral vitamin D_3_ or identical placebo for 90 days. Blood and ASL samples were collected preintervention and postintervention. 105 participants were originally enrolled, 86 completed the trial, and due to low protein concentration in the samples, 40 participants were finally analysed. Our primary outcome was ASL antimicrobial activity. We also considered secondary outcomes including changes in serum concentration of 25-hydroxyvitamin D_3_ (25(OH)D_3_), 1,25-hydroxyvitamin D_3_, calcium and parathyroid hormone (PTH). In addition, we studied the effect of cigarette smoke extract (CSE) exposure to primary human airway epithelial cell cultures on the gene expression of cathelicidin in response to vitamin D_3_ and expression of CYP27B1 (1-alpha hydroxylase), responsible for vitamin D_3_ activation.

**Results:**

Vitamin D_3_ supplementation significantly increased both ASL antimicrobial activity and serum concentration of 25(OH)D_3_. In a subgroup analysis, we found that smokers did not increase their baseline antimicrobial activity in response to vitamin D_3_. Exposure to CSE on human airway epithelia decreased baseline CYP27B1 gene expression and cathelicidin response to 25(OH)D_3_.

**Conclusion:**

Vitamin D_3_ supplementation for 90 days increases ASL antimicrobial activity. Data from this preliminary study suggest that smoking may alter the ability of airway epithelia to activate vitamin D_3_ and increase the gene expression of cathelicidin antimicrobial peptide.

**Trial registration number:**

NCT01967628; Post-results.

**Strengths and limitations of this study**

- We used airway surface liquid samples obtained through bronchoscopy from the same human subjects before and after interventions.
- The samples used are from a double-blind, randomised, placebo-controlled trial.
- We had a small number of samples in the subgroup analysis (smoking vs non-smoking group) that will require further confirmation in larger studies.

## Introduction

Vitamin D_3_ is a liposoluble vitamin naturally present in very few dietary sources and generated by the skin when exposed to sunlight. Despite supplementing food with vitamin D, a great proportion of the world’s population is still deficient.^1–3^ Vitamin D deficiency is found more often in smokers with accelerated lung function decline and in patients with severe chronic obstructive pulmonary disease (COPD).^4–8^ One of the proposed mechanisms for lung function decline and development of chronic respiratory diseases is the occurrence of airway infections.^9 10^ Since low vitamin D_3_ levels increase susceptibility to respiratory infections in both smokers and non-smokers,^11–14^ it is critical to investigate the effect of vitamin D supplementation on airway antimicrobial activity in both populations.

Vitamin D_3_ in the bloodstream is hydroxylated by the liver into 25-hydroxyvitamin D_3_ (25(OH)D_3_). This form is used conventionally to measure vitamin D_3_ sufficiency. Thereafter, the 1α-hydroxylase, constitutively active in the kidney, activated macrophages and in airway epithelia converts 25(OH)D_3_ into 1,25-dihydroxyvitamin D_3_ (1,25(OH)_2_D_3_).^15^ The active form of vitamin D_3_ binds to the intracellular vitamin D receptor to activate vitamin D response elements to modulate the gene expression of multiple pathways involved in serum calcium metabolism and bone remodelling.^16^ It also upregulates the expression of antimicrobial peptides (AMPs) present in the airway surface liquid (ASL), such as human cathelicidin (LL-37) and human beta defensins (hBDs) secreted by airway epithelia and immune cells.^15 17–19^ AMPs can kill pathogens within minutes as a first line of defence and their impairment is associated with the development of airway disease.^20^ Various mechanisms can impair AMP activity, such as: decreased production, decreased pH, increased ionic strength, increased host and pathogen proteases and increased polymerisation of AMPs with DNA/filamentous-actin.^21–26^

We hypothesised that vitamin D_3_ supplementation would increase human ASL antimicrobial activity in vivo. To test this hypothesis, we investigated the antimicrobial activity of biobanked ASL samples collected through bronchoscopy of a cohort of smoking and non-smoking participants that randomly received either 1000 international units (IU) of vitamin D_3_/day or matching placebo for 90 days.^27^ We also investigated the effect of smoking in the airway response to vitamin D_3 _in vivo and in vitro.

## Methods

### Study design

This study design has been previously published.^27^ Briefly, this is a single-centre, community-based, randomised, placebo-controlled double-blind trial. This pilot study investigated the effects of vitamin D_3_ supplementation on the innate immunity of the lung. The University of Iowa Hospital and Clinics Institutional Review Board approved the trial (IRB# 200607708). Written consent was obtained from all the subjects. This trial is registered on ClinicalTrials.gov (NCT01967628).

### Participants

**Figure 1:**
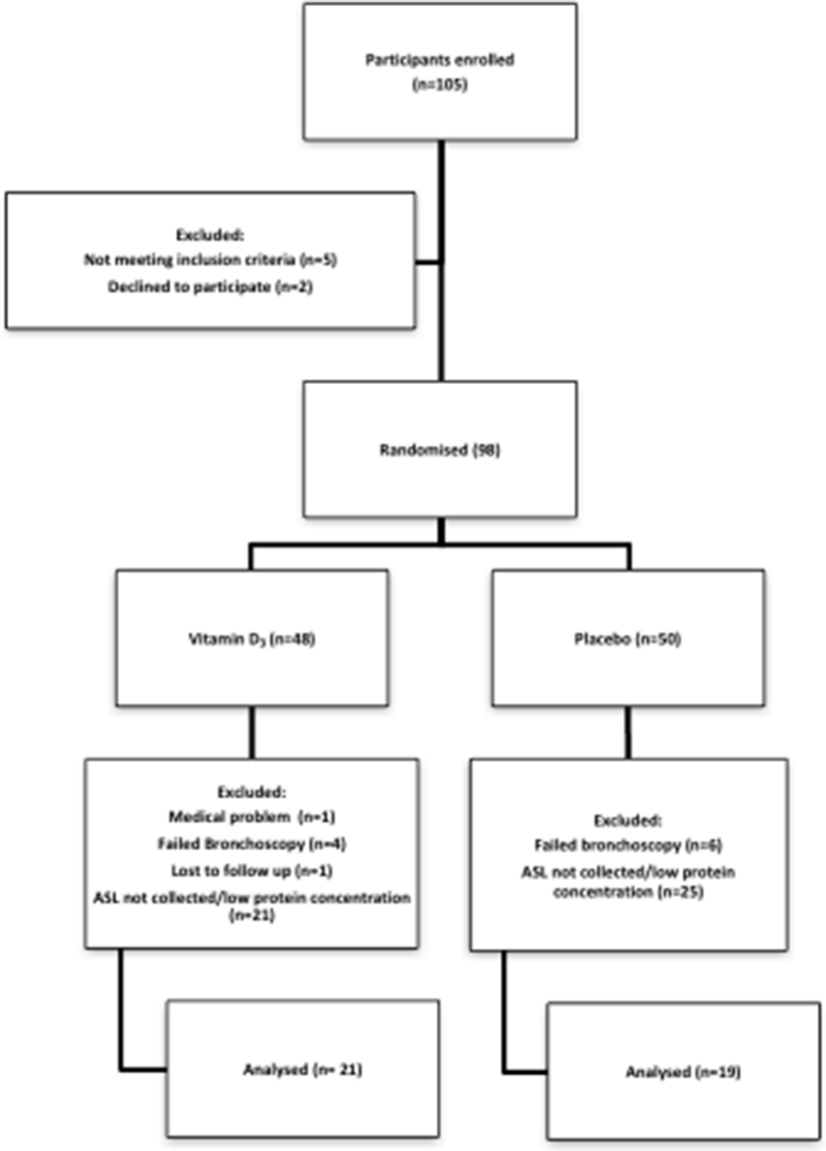
Participant flow diagram.

We recruited subjects 18–60 years old from the Iowa City community. We excluded participants if they were taking vitamin supplements within the previous 3 months, had any history of positive tuberculin test, pneumonia within 3 years, airway infection or antibiotic use within 6 weeks or vaccination within 1 month of enrolment. Additional exclusion criteria included any prescription medication except: oral contraceptives, topical medications, selected antidepressants, levothyroxine, acid reflux treatment, over-the-counter antihistamine or sleep aids. Participants were also excluded if they had one of these conditions: pregnancy, breastfeeding, asthma, diabetes, heart disease or allergy to lidocaine. We also excluded subjects that did not complete their second visit or if ASL collection was not possible due to technical limitations during bronchoscopy (figure 1).

### Interventions and randomisation

After informed consent was signed and pregnancy was excluded, the subject’s blood was drawn to assess 25(OH)D_3_, 1,25(OH)_2_D_3_, parathyroid hormone (PTH) and calcium serum concentration. Thereafter, a bronchoscopy was performed at the University of Iowa Hospitals and Clinics (Iowa City, Iowa, USA) as previously described.^27^ To collect the ASL, three sponges were placed, one at a time, on the right main stem bronchus or bronchus intermedius for 30 s and then eluted with 1 mL of isotonic saline into a 1.5 mL tube. The tube was vortexed for 1 min and the sponges removed. The samples were stored at −80°C. After collections, participants were randomised using a computer-generated list to receive either vitamin D_3_ (1000 IU/day, 90 000 IU total) or matching placebo. Study participants, the clinical research team and the sponsoring agency were blinded to intervention assignments.

### Outcomes

The primary outcome analysed for this subanalysis was ASL antimicrobial activity preintervention and postintervention (vitamin D_3_ or placebo). We considered secondary outcome changes in serum concentration of 25(OH)vitamin D_3_ (25(OH)D_3_), 1,25(OH)_2_D_3_, calcium and PTH. In a post hoc analysis, we also investigated the difference between the smoking and non-smoking groups in response to vitamin D_3_.

### ASL protein concentration

Briefly, aliquots were thawed at 4°C and total protein concentration was measured by a Bradford assay, interpolating samples to a bovine serum albumin standard curve. We adjusted sample concentrations with normal saline to 100 µg/mL of total protein. This threshold was selected prior the antimicrobial activity assay based on preliminary antimicrobial activity studies to minimise sample dilution and yet still be able to include the majority of the subjects.

### ASL antimicrobial activity

We added 10 µL of protein-corrected ASL (100 µg/mL) or protein uncorrected into a 96-well plate. Then, samples were challenged with 10 µL of a solution containing bioluminescent *Staphylococcus aureus* Xen 29 (~5×10^6^ colony forming units) injected by the precision peristaltic pump in the luminometer. After 4 min, we measured relative light units (RLU) as a surrogate for live bacteria as previously described.^21^ To minimise observer bias, we randomised the samples onto the plate. The luminometer injected the bacteria and measured RLU automatically, and the conditions were not revealed until the experiment was completed.

### Macrophage microarray

RNA preparation, quality analysis and microarray analysis were done as previously described.^28^ Measurements of genome-wide macrophage messenger RNA expression and analysis were done as previously described^27^ (Gene Expression Omnibus (GEO) accession # GSE56583).

### Human airway epithelia exposure to cigarette smoke extract

Differentiated primary human airway epithelia were cultured at the air liquid interface as previously described.^29^ We switched cultures to minimum essential media, with and without 25(OH)D_3_ (10^-7^ M) or 1,25(OH)_2_D_3_ (10^−7^ M) in the presence or absence of 3% cigarette smoke extract (CSE).^7 15^ We changed the media every day for the duration of the experiment and analysed by quantitative (q)PCR the relative gene expression of CYP27B1 which codes for 1α-hydroxylase and cathelicidin antimicrobial peptide (CAMP) which codes for the cationic antimicrobial peptide LL-37. First, we exposed cells to 25(OH)D_3_ or 1,25(OH)_2_D_3_ or CSE alone for 4 days and quantified the relative gene expression of CAMP and CYP27B1 (n=4). In separate experiments, we analysed the response to 25(OH)D_3_ (n=8) or 1,25(OH)_2_D_3_ (n=5) in epithelia exposed overnight to CSE or control and analysed the relative gene expression of CAMP by qPCR.

### Compliance

In the vitamin D_3_ group, 46% of subjects were compliant and 39% had residual doses. The mean number of missed capsules was approximately 6.5 capsules (range 1–15) for an average of total missed vitamin D_3_ of 6500 IU per subject. Four treated subjects did not bring back their pill bottles for compliance analysis. In the placebo group, 43% of subjects completed full therapy and 53% had missed doses. The mean number of missed capsules was 8.8 (range 1–30). One placebo subject did not bring back the pill bottle for analysis.

### Statistical analysis

Data are expressed as mean ± SEM For ASL antimicrobial activity, we used raw RLU and for the LL-37 experiments we normalised data as per cent of control using this formula:

$$Per\;cent\;of\;live\;bacteria=\frac{RLU\;from\;sample}{RLU\;from\;controlvehicle}\times 100$$

To determine the statistical significance between preintervention and postintervention (vitamin D_3_ and placebo), we used paired t-tests. We used multiple comparison analysis of variance (ANOVA) and Kruskal-Wallis to compare three or more conditions in the antibody treated ASL and LL-37. We also used Pearson test to calculate correlation coefficients. We did not exclude improbable data. Data analysis was performed using GraphPad Prism V.6.00 (GraphPad Software, California, USA). Power analysis were performed using G*Power V.3.1.9.2 (Germany).

## Results

### Participant demographics

We first analysed the differences in demographics between subjects receiving vitamin D_3_ or placebo (table 1). The vitamin D_3_ group had slightly more percentage of white, male and non-smoking participants compared with the placebo group. We found no significant differences in the age and number of pack-years in smokers between  vitamin D_3_ and placebo groups. We also analysed the differences in serum concentration of 25(OH)D_3_, 1,25(OH)_2_D_3_, calcium and PTH before intervention between the vitamin D_3_ and placebo groups and found no significant differences (table 2).

**Table 1:** Comparison of patient characteristics at baseline by treatment group

Subject characteristics	Vitamin D_3_	Placebo	p Value*
Number of participants	21	19	NA
Age (years)	25.8 (7.6)	29.4 (13)	0.2865
Male gender (%)	71.4	57.9	NA
Ethnicity (% white)	95.2	84.2	NA
Smokers (%)	23.8	36.8	NA
Pack/years	17.5 (9.1)	23 (12.7)	0.4272

Data expressed as mean and SD.

*Compared by unpaired t-test.

NA, not applicable.

**Table 2:** Treatment effect on several serum markers of vitamin D metabolism

	Pre vitamin D_3_	Post vitamin D_3_	p Value: previtamin D_3_ versus postvitamin D_3_*	Preplacebo	Postplacebo	p Value: preplacibo versus postplacebo*	p Value: previtamin D_3_ versus preplacebo^†^
25(OH)D_3_ (ng/mL)	29.9 (11.8)	34.38 (9.5)	0.0461	32.4 (18.1)	33.47 (19.3)	0.5415	0.6013
1,25(OH)_2_D_3_ (pg/mL)	51.6 (21.4)	44.3 (16.8)	0.1705	48.7 (21.5)	50.95 (18.2)	0.6319	0.6678
Calcium (mg/dL)	9.12 (0.33)	9.21 (0.32)	0.3817	9.05 (0.38)	9.04 (0.33)	0.9489	0.5285
Parathyroid Hormone (pg/mL)	37.01 (14.5)	38 (12)	0.7232	34.02 (8.6)	41.01 (15.4)	0.1970	0.4391

Data expressed as mean and SD.

*Compared by ratio paired t-test.

†Compared by unpaired t-test.

### Effect of vitamin D_3_ on serum levels of canonical vitamin D_3_ metabolites

**Figure 2:**
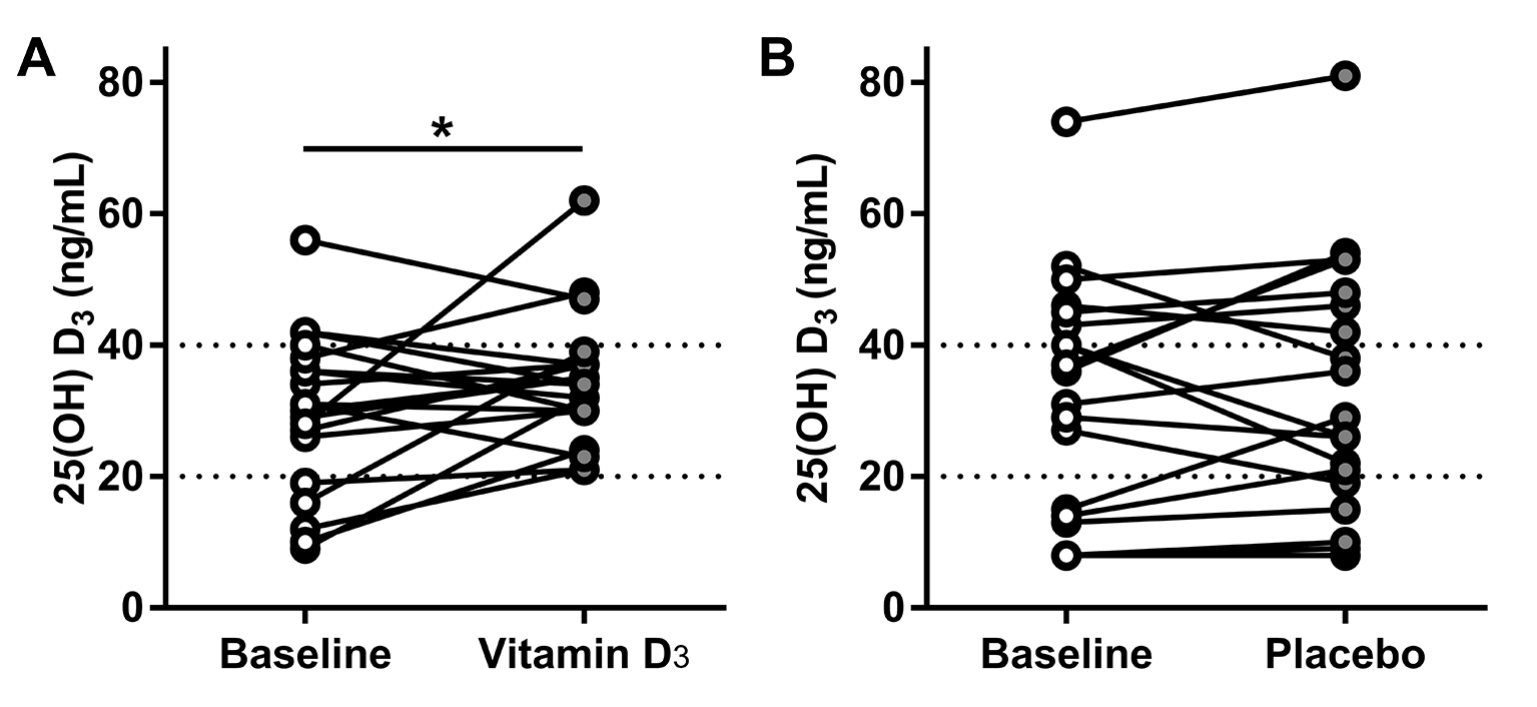
Treatment effect on serum concentration of 25(OH)D3. (A) Effect of vitamin D_3_ supplementation on serum concentration of 25(OH)D3 (*p=0.0461 by ratio paired t-test). (B) Effect of placebo supplementation on the serum concentration of 25(OH)D3. Dotted line at 20 ng/mL represents deficiency and above 40 represents optimal levels.

**Figure 3:**
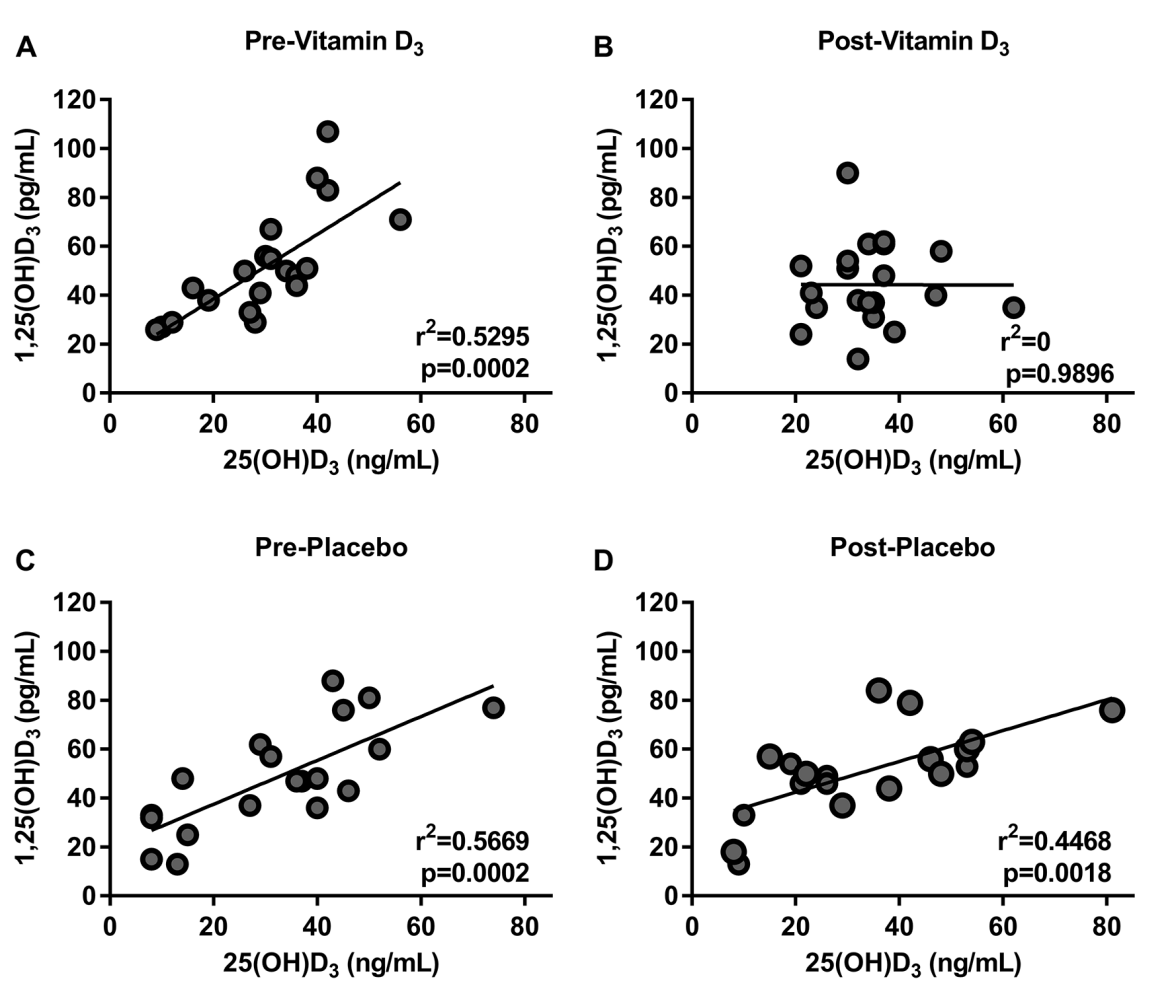
Vitamin D_3_ supplementation decreased the correlation of 25(OH)D3 to 1,25(OH)2D3. Pearson correlation of 25(OH)D3 and 1,25(OH)2D3 at baseline (A) previtamin D_3_, (B) postvitamin D_3_, (C) preplacebo, (D) and postplacebo. At baseline, there was a good correlation between the levels of 25(OH)D3 and 1,25(OH)2D3 in all groups (A and B). After vitamin D_3_ supplementation, the correlation disappears, while the placebo maintained this positive correlation (C and D).

We investigated the effect of vitamin D_3_ or placebo on the serum concentration of several canonical vitamin D metabolites such as: 25(OH)D_3_, 1,25(OH)_2_D_3_, calcium and PTH (table 2). We found that most of the participants had 25(OH)D_3_ levels less than 40 ng/mL and that only participants taking vitamin D proportionally increased their baseline 25(OH)D_3_ levels (figure 2). There was no statistically significant difference in the serum concentration of the other metabolites measured in either intervention (table 2). We also found that there was a positive correlation between 25(OH)D_3_ and 1,25(OH)_2_D_3_ at baseline in both groups. After the intervention, this correlation persisted in the placebo group but it was absent after vitamin D_3_ supplementation consistent with a biological effect of vitamin D_3_ (figure 3).

### Vitamin D_3_ increases ASL antimicrobial activity

**Figure 4:**
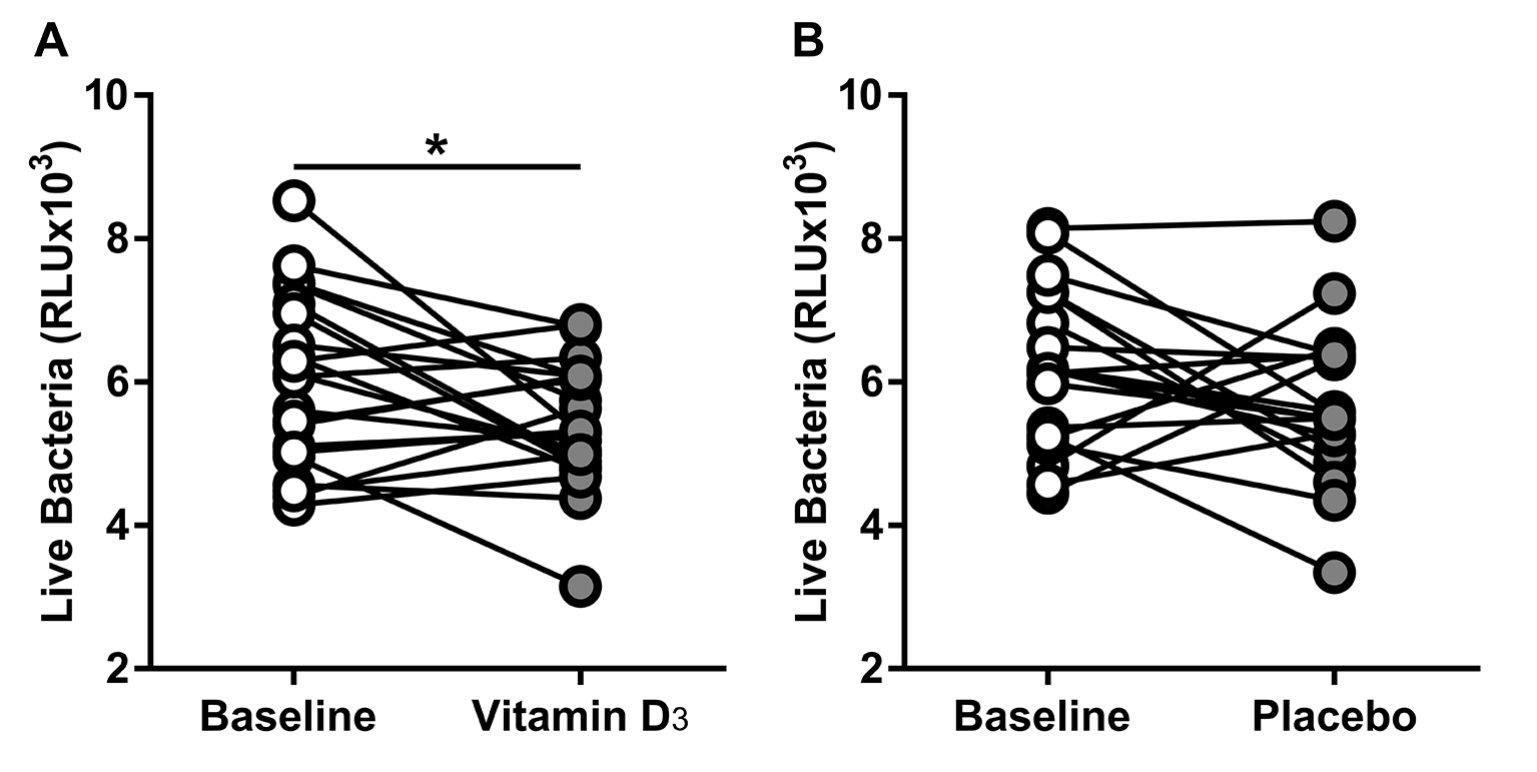
Vitamin D_3_ increased ASL antimicrobial activity. ASL antimicrobial activity assay. (A) ASL antimicrobial activity at baseline and after vitamin D_3_ (Baseline vs vitamin D mean±SD, 5980±1201 vs 5399±855.8; 95% CI of the difference −1122 to −40.37; *p=0.0365 by paired t-test). (B) ASL antimicrobial activity at baseline and after placebo. Placebo treatment did not increase the ASL antimicrobial activity compared to baseline (baseline vs placebo Mean±SD, 6155±1130 vs 5670±1089; 95% CI of the difference −1170 to 198.5; p=0.1532 by paired t-test). ASL, airway surface liquid; LL-37, human cathelicidin.

We investigated the effect of vitamin D_3_ supplementation on ASL antimicrobial activity using a bioluminescent bacterial challenge. We challenged ASL samples with bioluminescent bacteria and measured live bacteria by RLU after 2 min as a surrogate of antimicrobial activity. We interpreted a reduction in live bacteria after challenge in RLU as increased antimicrobial activity. We found that subjects taking vitamin D_3_ supplementation for 90 days had significantly lower live bacteria in RLU after challenge compared with their baseline (95% CI of the difference −1122 to −40.37; p=0.0365 by paired t-test) (figure 4A). Placebo treatment did not significantly decrease the number of live bacteria after challenge compared with baseline (95% CI of the difference −1170 to 198.5; p=0.1532 by paired t-test) (figure 4B). These results suggest that vitamin D_3_ supplementation for 90 days increases baseline ASL antimicrobial activity.

### 25(OH)D_3_ deficiency was associated with lower ASL antimicrobial activity

We hypothesised that subjects with serum vitamin D_3_ deficiency (<20 ng/mL) would have lower ASL antimicrobial activity compared with non-deficient subjects (≥20 ng/mL). To investigate the effect of vitamin D_3_ deficiency on ASL antimicrobial activity, we paired RLU values in the samples with their respective serum 25(OH)D_3_ regardless of allocation group (vitamin D_3_ or placebo) or whether they were baseline or postintervention samples. Thereafter, we compared the amount of live bacteria after challenge in the ASL samples with a clinically deficient 25(OH)D_3_ serum concentration to the samples with non-deficient levels. We found that ASL samples associated with vitamin D_3_ deficiency had significantly more live bacteria after challenge compared with non-deficient subjects (6394±289, n=16 vs 5646±129, n=64 respectively, p=0.0136 by unpaired t-test). These data suggest that vitamin D_3_ deficiency is associated with a decrease in ASL antimicrobial activity.

### LL-37 antibody inhibits ASL antimicrobial activity

We hypothesised that ASL antimicrobial activity is dependent on LL-37 activity. Since we were limited by volume and concentration of the samples, we took an indirect approach to investigate this hypothesis. We used an LL-37 antibody to neutralise the effect of cathelicidin, one of the antimicrobial peptides regulated by the vitamin D response elements, as previously described.^23 30 31^

**Figure 5:**
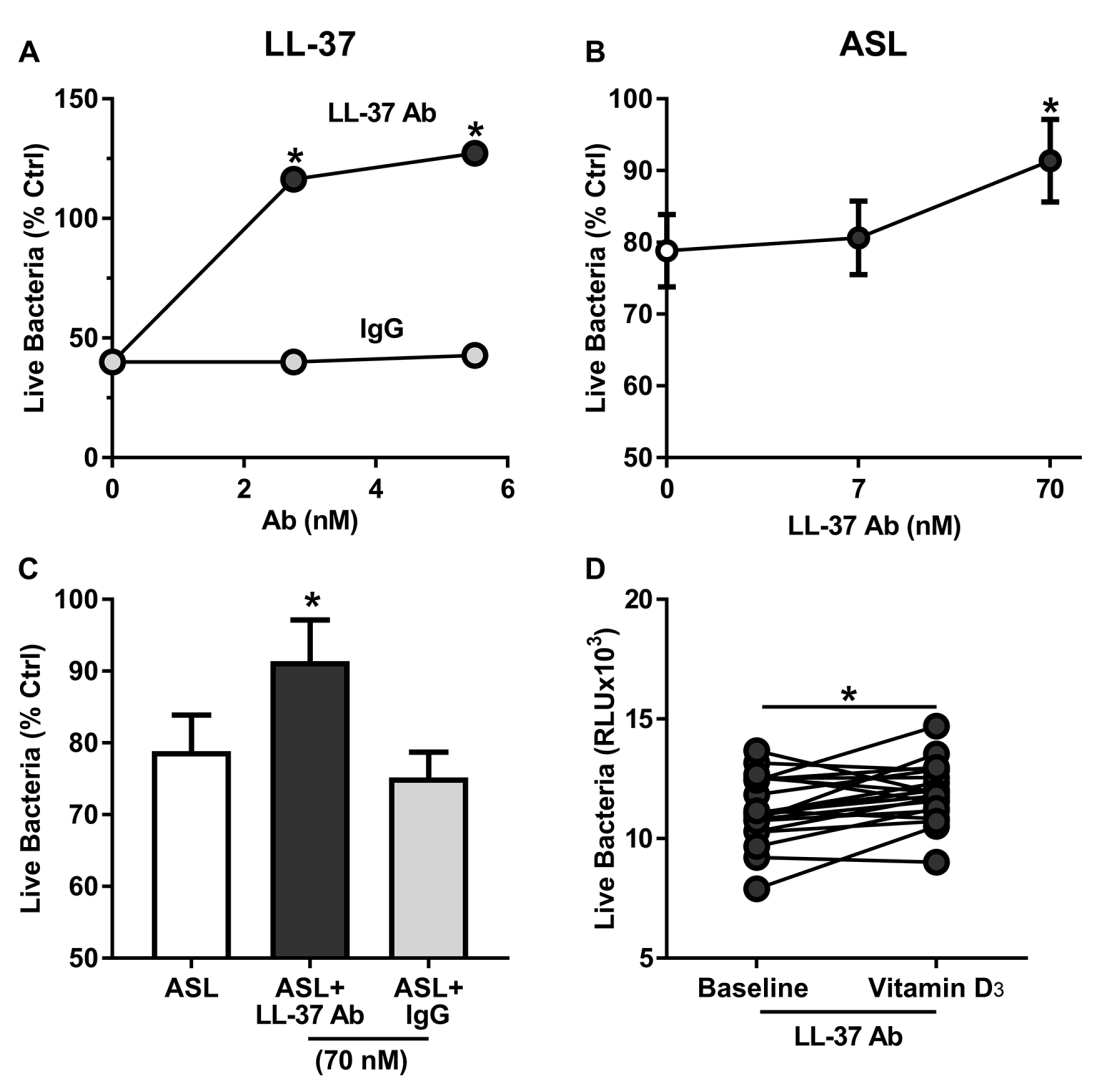
LL-37 antibody inhibits both LL-37 and ASL antimicrobial activity. (A) Antimicrobial activity assay of synthetic human LL-37 coincubated with increasing concentration of LL-37 antibody or IgG control (n=3). (B) Antimicrobial activity of ASL samples coincubated with increasing concentrations of LL-37 antibody (n=6). (C) ASL antimicrobial activity of ASL samples coincubated with LL-37 antibody (70 nM) and IgG (70 nM) as control (N=6; *p=<0.05 by multiple comparisons ANOVA). (D) Antimicrobial activity of ASL samples before and after vitamin D_3_ supplementation treated with an LL-37 antibody (N=21; *p=0.0196 by paired t-test). ANOVA, analysis of variance; ASL, airway surface liquid; LL-37, human cathelicidin.

When we coincubated increasing concentrations of LL-37 antibody with synthetic LL-37, we observed inhibition of the antimicrobial activity in a concentration-dependent manner compared with control antibody (goat IgG, antimouse IgG) (figure 5A). We also tested the effect of increasing doses of LL-37 antibody on seven random preintervention ASL samples from non-smokers, resulting in decreased ASL antimicrobial activity in a concentration-dependent manner (figure 5B). When we treated ASL samples with control antibody at maximal concentration, we observed no effect compared with ASL without the antibody at 4 min after bacterial challenge (figure 5C). These results suggest that LL-37 antibody can inhibit ASL antimicrobial activity.

### Anti-LL-37 blocks the vitamin D_3_-induced improvement of ASL antimicrobial activity

We hypothesised that LL-37 antibody would block the increased antimicrobial activity of the ASL samples from non-smokers postvitamin D_3_ supplementation. When we coincubated ASL samples previtamin D_3_ and postvitamin D_3_ with LL-37 antibody (70 nM), the increased antimicrobial activity was significantly reversed (figure 5D).

### Vitamin D_3_ does not increase cathelicidin gene expression in alveolar macrophages

AMPs in ASL can come from two main sources: inflammatory cells such as macrophages and airway epithelial cells. Since it has been reported that vitamin D_3_ increases LL-37 in both human macrophages and airway epithelia,^15 32^ we investigated the change in CAMP gene expression in human alveolar macrophages from smokers and non-smokers treated with vitamin D_3_. We found no significant difference in either smokers or non-smokers (fold change −1.05, p=0.33 in non-smokers and fold change −1.15, p=0.16 in smokers). These results suggest that airway epithelia might be responsible for the increase in ASL antimicrobial activity due to vitamin D_3_.

### 25(OH)D_3_ and 1,25(OH)_2_D_3_ increase airway CAMP gene expression

**Figure 7:**
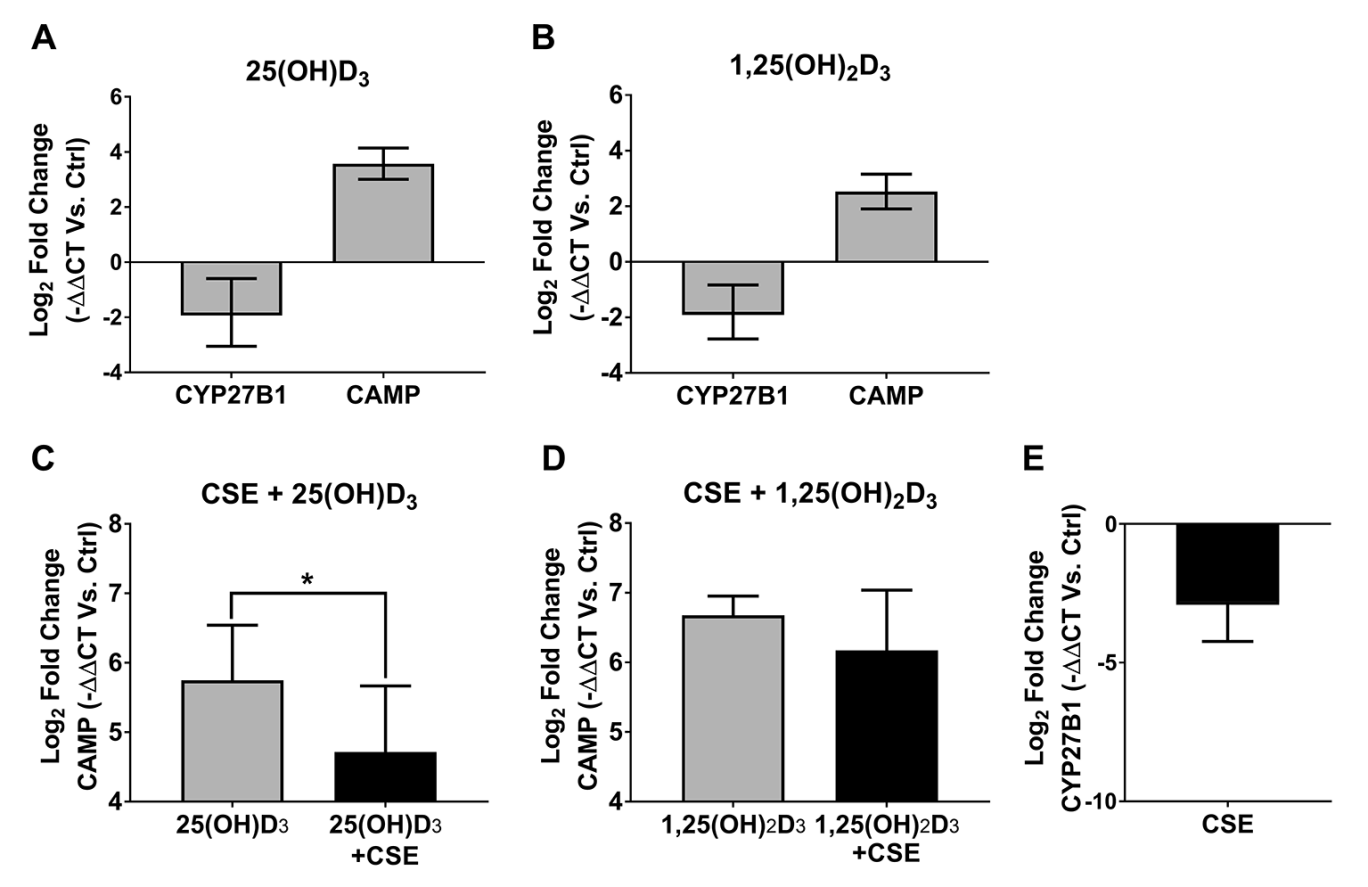
Human airway epithelia exposed to CSE had decreased gene expression of CAMP in response to 25(OH)D3 and decreased CYP27B1. (A and B) Gene expression of CYP27B1 and CAMP by qPCR of primary human airway epithelia exposed to 25(OH)-D3 (A) and 1,25(OH)2D3 (B). Both forms of vitamin D3 increased CAMP relative gene expression and decreased CYP27B1. (C) Gene expression of CAMP in response to 25(OH)D3. Exposure to CSE blunted the increase in CAMP in response to 25(OH)D3 (*p=0.0398 by paired t-test). (D) Gene expression of CAMP in response to 1,25(OH)2D3. Exposure to CSE did not affect the increase in CAMP in response to 1,25(OH)2D3. (E) Gene expression of CYP27B1 qPCR of primary human airway epithelia exposed to CSE. Exposure to CSE decreased the relative gene expression of CYP27B1. CAMP, cathelicidin antimicrobial peptide; CSE, cigarette smoke extract; qPCR, quantitative PCR.

Since we did not find an increase in CAMP gene expression in macrophages, we investigated the effect of vitamin D_3_ exposure in primary human airway epithelial cells cultured at the air–liquid interface. As expected, supplementing the media with 25(OH)D_3_ and 1,25(OH)_2_D_3_ increased the relative gene expression of CAMP. In addition, vitamin D_3_ supplementation decreased CYP27B1, which encodes 1-alpha hydroxylase, the enzyme that converts vitamin D_3_ into its active form (figure 7A,B).^15^ This decrease in CYP27B1 is consistent with a self-regulatory loop that decreases excessive vitamin D_3_ activation.^15 33^

### Smokers did not increase ASL antimicrobial activity

**Figure 6:**
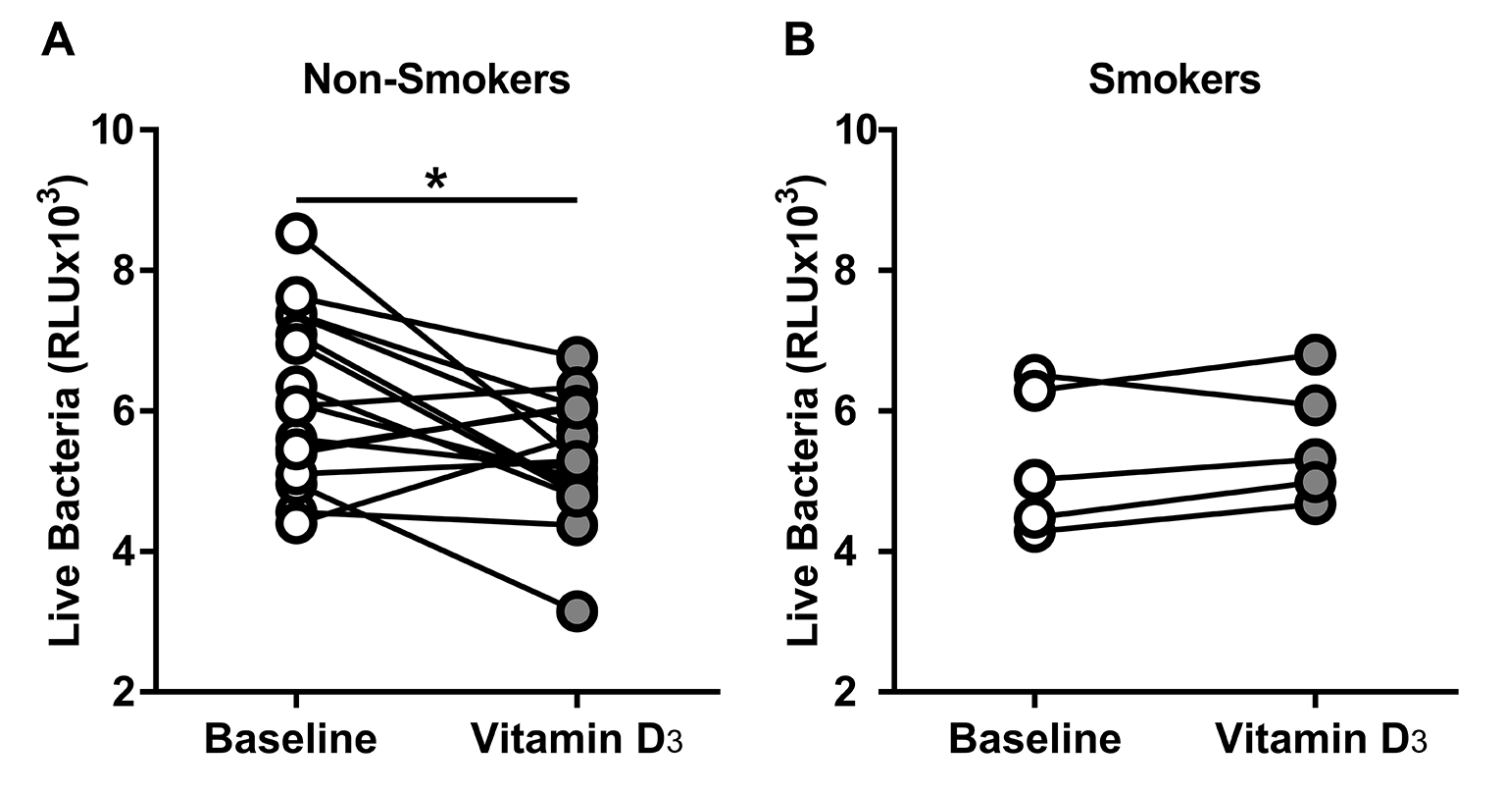
Vitamin D_3_ increased ASL antimicrobial activity in non-smokers. ASL antimicrobial activity assay. (A) Non-smokers ASL antimicrobial activity at baseline and after vitamin D_3_. Non-smokers significantly increased their baseline antimicrobial activity (baseline vs vitamin D_3_ mean±SD, 6186±1205 vs 5344±874.3; 95% CI of the difference −1502 to −181.9; *p=0.0159 by paired t-test). (B) Smokers ASL antimicrobial activity at baseline and after vitamin D_3_. Smokers did not increase their ASL antimicrobial activity (baseline vs vitamin D_3_ mean±SD, 5321±1029 vs 5575±863; 95% CI of the difference −235.1 to 743.2; p=0.2227 by paired t-test). ASL, airway surface liquid.

In a post hoc analysis, we investigated the effect of smoking in the antimicrobial activity of the ASL. When we analysed the ASL from smokers and the non-smokers, only the non-smokers significantly decreased the number of live bacteria compared with their baseline (baseline vs vitamin D_3_ in non-smokers) (figure 6A). Although the smokers had a small sample size, we did not observe any difference (figure 6B). Using the data from the non-smokers that received vitamin D_3_ we determined an effect size of 0.8462, and using a power of 0.8 and alpha of 0.05, we calculated an a priori sample size of at least 14 subjects to detect a difference in the smokers.

### CSE decreased CAMP gene expression response to vitamin D_3_

Since smokers did not improve their baseline antimicrobial activity when supplemented with vitamin D_3_, we investigated the effect of CSE exposure on CAMP gene expression in response to 25(OH)D_3 _in vitro. We found that airway epithelial cells exposed to CSE and supplemented with 25(OH)D_3_ had a smaller increase in CAMP gene expression compared with unexposed cells (figure 7C). We then investigated if supplementing 1,25(OH)_2_D_3_ would overcome this decreased response in CAMP gene expression in CSE-exposed human airway epithelial cells. We found a similar increase in CAMP gene expression in both CSE-exposed and unexposed human airway epithelial cells in response to 1,25(OH)_2_D_3_ (figure 7D). We conclude that CSE exposure decreases cathelicidin gene expression in response to 25(OH)D_3._ This can be overcome by supplementing 1,25(OH)_2_D_3_, suggesting that CSE might affect the activation of vitamin D_3_.

### CSE decreases airway CYP27B1 gene expression

We hypothesised that exposure to cigarette smoke decreases the gene expression of 1-alpha hydroxylase, responsible for the activation of 25(OH)D_3_. To test this hypothesis, we exposed human airway epithelial cells to CSE and analysed the gene expression of CYP27B1 by qPCR. We found that exposure to CSE decreased CYP27B1 gene expression compared with unexposed cells (figure 7E). This result suggests that cigarette smoke exposure might decrease the activation of vitamin D_3_.

## Discussion

Prevention of respiratory infections by vitamin D_3_ supplementation is controversial. Some reports show that vitamin D_3_ supplementation reduces the risk of developing respiratory infections^34^ while others do not show such protection.^35^ To our knowledge, this is the first in vivo study demonstrating that vitamin D_3_ supplementation increases baseline human ASL antimicrobial activity using a standardised sample and inoculum.

In our cohort, vitamin D_3_ supplementation for 90 days increased 25(OH)D_3_ serum concentration and increased ASL antimicrobial activity. To explain the later result, we presume that as calcium concentration increases several negative feedback loops tightly regulate vitamin D_3_ activation. For example, increased 1,25(OH)_2_D_3_ concentrations decreases 1-α hydroxylase and increases 24-hydroxylase, which is responsible for inactivation of both 25(OH)D_3_ and 1,25(OH)_2_D_3_.^33^

In addition, we found that ASL samples matched with a deficient vitamin D_3_ serum concentration had significantly lower antimicrobial activity compared with samples paired with sufficient vitamin D_3_. These results are consistent with epidemiological studies that report that low vitamin D_3_ levels increase the susceptibility to respiratory infections.^11–14^

We speculate that vitamin D_3_ increases the ASL antimicrobial activity by upregulating gene expression of antimicrobial peptides like cathelicidins.^15 17–19^ Since we did not find any difference in CAMP gene expression in macrophages, we hypothesised that the airway epithelium was responsible for the increase in ASL antimicrobial activity due to vitamin D_3_. Since we were limited by the volume and concentration of the samples, we took an indirect approach to confirm our hypothesis. As a proof of concept, we used an LL-37 antibody to neutralise the effect of LL-37 in the ASL.^23 30^ We found that the improvement in ASL antimicrobial activity after vitamin D_3_ supplementation was reversed when the ASL was treated with an LL-37 antibody. This effect can be in part due to both functional inhibition of this antimicrobial peptide and/or decreased synergism between LL-37 and other antimicrobial peptides such as hBDs.^22^ Future studies should consider sampling more volume of ASL to be able to measure directly AMPs. Nonetheless, He *et al* found that Vitamin D_3_ supplementation for 14 weeks increased the concentration of cathelicidin in both plasma and saliva in healthy subjects.^36^

We also did a subgroup analysis on the subjects who were smokers, and found compelling results that suggest that they did not increase their ASL antimicrobial activity after vitamin D_3_ supplementation. We acknowledge that our sample size was small and we might be underpowered to detect the difference. However, our in vitro study showing that human airway epithelial cells exposed to CSE in vitro had decreased cathelicidin gene expression in response to 25(OH)D_3_ strongly supports the feasibility of our in vivo finding, despite lack of power. In addition, it has been reported that cigarette smoke exposure is negatively correlated with LL-37 in human saliva.^37^ Furthermore, patients with COPD who have high risk of frequent exacerbations also have decrea*s*ed plasma levels of LL-37 compared with control subjects.^38^ These results warrant further evaluation in larger sample sizes.

Our in vitro results also suggest that one of the mechanisms responsible for this finding is that cigarette smoke reduces gene expression coding for the enzyme responsible for converting vitamin D_3_ into its active form. This is consistent with a prior report that exposure to cigarette smoke was associated with impaired ability of human airway epithelia to hydroxylate 25(OH)D_3_ to 1,25(OH)_2_D_3_.^7^ When we used the active form of vitamin D_3_, we found a similar increase in cathelicidin gene expression regardless of CSE exposure. Heulens *et al* also reported that human macrophages exposed to CSE had a similar increase in cathelicidin gene expression in response to 1,25(OH)_2_D_3_compared with unexposed cells.^39^

Given that cigarette smoke exposure (1) decreased cathelicidin gene expression in response to 25(OH)D_3_, (2) decreased CYP27B1 gene expression and (3) did not affect the cathelicidin gene expression response to active vitamin D_3_, we propose that one mechanism by which CSE impairs airway innate immunity is by decreasing local vitamin D_3_ activation.

Another plausible explanation for decreased response to vitamin D_3_ in the smokers is different processing of hCAP18, the parent molecule of LL-37 before being processed or LL-37 itself. Smoking increases the concentration of proteolytic proteins such as cathepsin D and neutrophils elastase.^40 41^ Proteolysis by these enzymes can decrease LL-37 in the airways and its antimicrobial activity.^23^

Furthermore, although cathelicidin is a relevant antimicrobial peptide, other antimicrobials that have a vitamin D response element like hBDs might be also increased by vitamin D_3_ supplementation.^42^ Herr *et al* reported that airway epithelium exposed to smoke significantly reduced hBD-2 and antimicrobial activity in vitro. In addition, former or current smokers with pneumonia had decreased concentration of hBD-2 in pharyngeal washing fluid compared with non-smokers.^43^

Our study has several strengths, including analysing ASL samples from the same subjects before and after randomised intervention. We also recognise that we have several limitations such as the small number of samples in the subgroup analysis and the indirect approach to assess the role of LL-37 in the ASL antimicrobial activity using a neutralising antibody. Finally, it is conceivable that smokers require even higher doses of vitamin D_3_ and/or a longer duration of treatment.

### Conclusions

We provide evidence that vitamin D_3_ supplementation for 90 days increases ASL antimicrobial activity in non-smoking humans. We presume that this effect occurs via an increase in ASL AMPs. Conversely, smoking participants did not improve their ASL antimicrobial activity, and we propose that the mechanism is, in part, due to impaired conversion of vitamin D_3_ to its active form leading to a decreased expression of airway AMPs. Future studies with a higher number of subjects are required to confirm the effect modification of smoking in the ASL antimicrobial activity in response to vitamin D_3 _in vivo, as well as the effect of active vitamin D_3_ in the airway antimicrobial activity of smokers.

10.1136/bmjresp-2017-000211.supp1 Supplementary data

10.1136/bmjresp-2017-000211.supp2 Supplementary data

10.1136/bmjresp-2017-000211.supp3 Supplementary data

## References

[1] Cashman KD, Dowling KG, Škrabáková Z, et al Vitamin D deficiency in Europe: pandemic? Am J Clin Nutr 2016;103:1033–44.doi:10.3945/ajcn.115.120873 26864360 10.3945/ajcn.115.120873 PMC5527850

[2] Akkermans MD, van der Horst-Graat JM, Eussen SR, et al Iron and vitamin D deficiency in healthy young children in Western Europe despite Current Nutritional Recommendations. J Pediatr Gastroenterol Nutr 2016;62:635–42.doi:10.1097/MPG.0000000000001015 26488124 10.1097/MPG.0000000000001015

[3] Ng K, Scott JB, Drake BF, et al Dose response to vitamin D supplementation in african Americans: results of a 4-arm, randomized, placebo-controlled trial. Am J Clin Nutr 2014;99:587–98.doi:10.3945/ajcn.113.067777 24368437 10.3945/ajcn.113.067777 PMC3927692

[4] Cutillas-Marco E, Fuertes-Prosper A, Grant WB, et al Vitamin D deficiency in South Europe: effect of smoking and aging. Photodermatol Photoimmunol Photomed 2012;28:159–61.doi:10.1111/j.1600-0781.2012.00649.x 22548399 10.1111/j.1600-0781.2012.00649.x

[5] Lange NE, Sparrow D, Vokonas P, et al Vitamin D deficiency, smoking, and lung function in the normative aging study. Am J Respir Crit Care Med 2012;186:616–21.doi:10.1164/rccm.201110-1868OC 22822023 10.1164/rccm.201110-1868OC PMC3480523

[6] Larose TL, Chen Y, Camargo CA, et al Factors associated with vitamin D deficiency in a norwegian population: the HUNT Study. J Epidemiol Community Health 2014;68:165–70.doi:10.1136/jech-2013-202587 24197920 10.1136/jech-2013-202587

[7] Mulligan JK, Nagel W, O'Connell BP, et al Cigarette smoke exposure is associated with vitamin D3 deficiencies in patients with chronic rhinosinusitis. J Allergy Clin Immunol 2014;134:342–9.doi:10.1016/j.jaci.2014.01.039 24698317 10.1016/j.jaci.2014.01.039

[8] Zhu B, Zhu B, Xiao C, et al Vitamin D deficiency is associated with the severity of COPD: a systematic review and meta-analysis. Int J Chron Obstruct Pulmon Dis 2015;10:1907–16.doi:10.2147/COPD.S89763 26392765 10.2147/COPD.S89763 PMC4574800

[9] Hansbro PM, Starkey MR, Mattes J, et al Pulmonary immunity during respiratory infections in early life and the development of severe asthma. Ann Am Thorac Soc 2014;11(Suppl 5):S297–302.doi:10.1513/AnnalsATS.201402-086AW 25525736 10.1513/AnnalsATS.201402-086AW

[10] Hayden LP, Hobbs BD, Cohen RT, et al Childhood pneumonia increases risk for chronic obstructive pulmonary disease: the COPDGene study. Respir Res 2015;16:115 doi:10.1186/s12931-015-0273-8 26392057 10.1186/s12931-015-0273-8 PMC4578796

[11] Leis KS, McNally JD, Montgomery MR, et al Vitamin D intake in young children with acute lower respiratory infection. Transl Pediatr 2012;1:6–14.doi:10.3978/j.issn.2224-4336.2011.11.01 26835258 10.3978/j.issn.2224-4336.2011.11.01 PMC4728844

[12] Ginde AA, Mansbach JM, Camargo CA Association between serum 25-hydroxyvitamin D level and upper respiratory tract infection in the Third National Health and Nutrition Examination Survey. Arch Intern Med 2009;169:384–90.doi:10.1001/archinternmed.2008.560 19237723 10.1001/archinternmed.2008.560 PMC3447082

[13] Quraishi SA, Bittner EA, Christopher KB, et al Vitamin D status and community-acquired pneumonia: results from the Third National Health and Nutrition Examination Survey. PLoS One 2013;8:e81120 doi:10.1371/journal.pone.0081120 24260547 10.1371/journal.pone.0081120 PMC3829945

[14] Bowler RP, Kim V, Regan E, et al Prediction of acute respiratory disease in current and former smokers with and without COPD. Chest 2014;146:941–50.doi:10.1378/chest.13-2946 24945159 10.1378/chest.13-2946 PMC4188150

[15] Hansdottir S, Monick MM, Hinde SL, et al Respiratory epithelial cells convert inactive vitamin D to its active form: potential effects on host defense. J Immunol 2008;181:7090–9.doi:10.4049/jimmunol.181.10.7090 18981129 10.4049/jimmunol.181.10.7090 PMC2596683

[16] Holick MF Vitamin D deficiency. N Engl J Med 2007;357:266–81.doi:10.1056/NEJMra070553 17634462 10.1056/NEJMra070553

[17] Wang TT, Nestel FP, Bourdeau V, et al Cutting edge: 1,25-dihydroxyvitamin D3 is a direct inducer of antimicrobial peptide gene expression. J Immunol 2004;173:2909–12.doi:10.4049/jimmunol.173.5.2909 15322146 10.4049/jimmunol.173.5.2909

[18] Janssens W, Decramer M, Mathieu C, et al Vitamin D and chronic obstructive pulmonary disease: hype or reality? Lancet Respir Med 2013;1:804–12.doi:10.1016/S2213-2600(13)70102-4 24461760 10.1016/S2213-2600(13)70102-4

[19] Gombart AF, Borregaard N, Koeffler HP Human cathelicidin antimicrobial peptide (CAMP) gene is a direct target of the vitamin D receptor and is strongly up-regulated in myeloid cells by 1,25-dihydroxyvitamin D3. Faseb J 2005;19:1067–77.doi:10.1096/fj.04-3284com 15985530 10.1096/fj.04-3284com

[20] Stoltz DA, Meyerholz DK, Welsh MJ Origins of cystic fibrosis lung disease. N Engl J Med 2015;372:351–62.doi:10.1056/NEJMra1300109 25607428 10.1056/NEJMra1300109 PMC4916857

[21] Travis SM, Conway BA, Zabner J, et al Activity of abundant antimicrobials of the human airway. Am J Respir Cell Mol Biol 1999;20:872–9.doi:10.1165/ajrcmb.20.5.3572 10226057 10.1165/ajrcmb.20.5.3572

[22] Abou Alaiwa MH, Reznikov LR, Gansemer ND, et al pH modulates the activity and synergism of the airway surface liquid antimicrobials β-defensin-3 and LL-37. Proc Natl Acad Sci USA 2014;111:18703–8.doi:10.1073/pnas.1422091112 25512526 10.1073/pnas.1422091112 PMC4284593

[23] Bergsson G, Reeves EP, McNally P, et al LL-37 complexation with glycosaminoglycans in cystic fibrosis lungs inhibits antimicrobial activity, which can be restored by hypertonic saline. J Immunol 2009;183:543–51.doi:10.4049/jimmunol.0803959 19542465 10.4049/jimmunol.0803959

[24] Bucki R, Byfield FJ, Janmey PA Release of the antimicrobial peptide LL-37 from DNA/F-actin bundles in cystic fibrosis sputum. Eur Respir J 2007;29:624–32.doi:10.1183/09031936.00080806 17215317 10.1183/09031936.00080806

[25] Sieprawska-Lupa M, Mydel P, Krawczyk K, et al Degradation of human antimicrobial peptide LL-37 by Staphylococcus aureus-derived proteinases. Antimicrob Agents Chemother 2004;48:4673–9.doi:10.1128/AAC.48.12.4673-4679.2004 15561843 10.1128/AAC.48.12.4673-4679.2004 PMC529204

[26] Rivas-Santiago B, Serrano CJ, Enciso-Moreno JA Susceptibility to infectious diseases based on antimicrobial peptide production. Infect Immun 2009;77:4690–5.doi:10.1128/IAI.01515-08 19703980 10.1128/IAI.01515-08 PMC2772553

[27] Gerke AK, Pezzulo AA, Tang F, et al Effects of vitamin D supplementation on alveolar macrophage gene expression: preliminary results of a randomized, controlled trial. Multidiscip Respir Med 2014;9:18 doi:10.1186/2049-6958-9-18 24669961 10.1186/2049-6958-9-18 PMC3986866

[28] Graff JW, Powers LS, Dickson AM, et al Cigarette smoking decreases global microRNA expression in human alveolar macrophages. PLoS One 2012;7:e44066 doi:10.1371/journal.pone.0044066 22952876 10.1371/journal.pone.0044066 PMC3430644

[29] Karp PH Epithelial cell culture protocols. Totowa, NJ: Humana Press, Inc, 2002.

[30] Heilborn JD, Nilsson MF, Kratz G, et al The cathelicidin anti-microbial peptide LL-37 is involved in re-epithelialization of human skin wounds and is lacking in chronic ulcer epithelium. J Invest Dermatol 2003;120:379–89.doi:10.1046/j.1523-1747.2003.12069.x 12603850 10.1046/j.1523-1747.2003.12069.x

[31] Krasnodembskaya A, Song Y, Fang X, et al Antibacterial effect of human mesenchymal stem cells is mediated in part from secretion of the antimicrobial peptide LL-37. Stem Cells 2010;28:2229–38.doi:10.1002/stem.544 20945332 10.1002/stem.544 PMC3293245

[32] Bagaitkar J, Demuth DR, Scott DA Tobacco use increases susceptibility to bacterial infection. Tob Induc Dis 2008;4:12 doi:10.1186/1617-9625-4-12 19094204 10.1186/1617-9625-4-12 PMC2628337

[33] Mazahery H, von Hurst PR Factors affecting 25-Hydroxyvitamin D concentration in response to vitamin D supplementation. Nutrients 2015;7:5111–42.doi:10.3390/nu7075111 26121531 10.3390/nu7075111 PMC4516990

[34] Martineau AR, Jolliffe DA, Hooper RL, et al Vitamin D supplementation to prevent acute respiratory tract infections: systematic review and meta-analysis of individual participant data. BMJ 2017;356:i6583 doi:10.1136/bmj.i6583 28202713 10.1136/bmj.i6583 PMC5310969

[35] Murdoch DR, Slow S, Chambers ST, et al Effect of vitamin D3 supplementation on upper respiratory tract infections in healthy adults: the VIDARIS randomized controlled trial. JAMA 2012;308:1333–9.doi:10.1001/jama.2012.12505 23032549 10.1001/jama.2012.12505

[36] He CS, Fraser WD, Tang J, et al The effect of 14 weeks of vitamin D3 supplementation on antimicrobial peptides and proteins in Athletes. J Sports Sci 2016;34:67–74.doi:10.1080/02640414.2015.1033642 25861808 10.1080/02640414.2015.1033642

[37] Takeuchi Y, Nagasawa T, Katagiri S, et al Salivary levels of antibacterial peptide (LL-37/hCAP-18) and cotinine in patients with chronic periodontitis. J Periodontol 2012;83:766–72.doi:10.1902/jop.2011.100767 21942788 10.1902/jop.2011.100767

[38] Yang YM, Guo YF, Zhang HS, et al Antimicrobial peptide LL-37 circulating levels in chronic obstructive pulmonary disease patients with high risk of frequent exacerbations. J Thorac Dis 2015;7:740–5.doi:10.3978/j.issn.2072-1439.2015.04.33 25973241 10.3978/j.issn.2072-1439.2015.04.33 PMC4419288

[39] Heulens N, Korf H, Mathyssen C, et al 1,25-Dihydroxyvitamin D modulates antibacterial and inflammatory response in human cigarette Smoke-Exposed macrophages. PLoS One 2016;11:e0160482 doi:10.1371/journal.pone.0160482 27513734 10.1371/journal.pone.0160482 PMC4981391

[40] Chang JC, Yoo OH, Lesser M Cathepsin D activity is increased in alveolar macrophages and bronchoalveolar lavage fluid of smokers. Am Rev Respir Dis 1989;140:958–60.doi:10.1164/ajrccm/140.4.958 2802382 10.1164/ajrccm/140.4.958

[41] Betsuyaku T, Nishimura M, Yoshioka A, et al Elastin-derived peptides and neutrophil elastase in bronchoalveolar lavage fluid. Am J Respir Crit Care Med 1996;154:720–4.doi:10.1164/ajrccm.154.3.8810611 8810611 10.1164/ajrccm.154.3.8810611

[42] Li P, Wu T, Su X, et al Activation of vitamin D regulates response of human bronchial epithelial cells to Aspergillus fumigatus in an autocrine fashion. Mediators Inflamm 2015;2015:1–14.doi:10.1155/2015/208491 10.1155/2015/208491 PMC4413954 25960612

[43] Herr C, Beisswenger C, Hess C, et al Suppression of pulmonary innate host defence in smokers. Thorax 2009;64:144–9.doi:10.1136/thx.2008.102681 18852155 10.1136/thx.2008.102681

