# Supplementary material for: Effect of vitamin D3 on the antimicrobial activity of human airway surface liquid: preliminary results of a randomised placebo-controlled double-blind trial
Source: BMJ Open Respir Res. 2017 Jun 4;4(1):e000211. doi: 10.1136/bmjresp-2017-000211 (PMC5531307; doi:10.1136/bmjresp-2017-000211)
Supplement: Supplementary data [file bmjresp-2017-000211supp001.pdf]

SUPPLEMENT FILE

Bronchoscopy

After informed consent was signed, women of childbearing age underwent urine pregnancy screen. Subjects were pre-medicated with atropine (0.6 mg IM) and either morphine (10 mg IM) or meperidine (12.5-25 mg IM). The airways were numbed using topical lidocaine (2-4%). A flexible bronchoscope (model P160 or P180; Olympus) was introduced trans-nasally or trans-orally into the right main bronchium under standard clinical monitoring.

Vitamin D_3 serum measurement

Samples were sent to the Laboratory at the University of Iowa Department of Pathology. $25(OH)D_3$ was measured by Electrochemiluminescence, Multiplex flow immunoassay, and $1,25(OH)_2D_3$ was measured by quantitative chemiluminescent immunoassay.

Bacterial strains

Staphylococcus aureus strain Xen 29® (Caliper LifeSciences Bioware™, MA, USA) was chosen because it is a human isolate strain that possesses a copy of the modified Photorhabdus luminescens luxABCDE operon at a single integration site on the bacterial chromosome. This feature allowed us to measure a thermostable real time luminescent signal in Relative Light Units (RLU). Xen 29® was grown from a bacterial glycerol stock on a tryptic soy agar plate containing kanamycin (0.2 mg/mL) overnight. On the next day, four to five colonies were grown in Tryptic Soy Broth medium (TSB) (BD Difco, New Jersey, USA) overnight. Thereafter, a subculture of log phase bacteria

was centrifuged (1 min, 25 °C, 16 RCF) and washed twice with Phosphate Buffered Saline with no calcium or magnesium (PBS -/-) (Gibco® Life Technologies, Massachusetts, USA). The final bacterial pellet was suspended in minimal media consisting of $NaPO_4$buffer supplemented with 100 mM NaCl and 1% TSB to sustain bacterial luminescence as previously described E[1].

ASL antimicrobial activity

We used a bioluminescent bacterial assay using *Staphylococcus aureus* Xen 29 (Caliper Biosciences). This assay allowed us to determine bacterial viability in a high throughput fashion and previous studies have determined a good correlation between RLU and Colony Forming Units (CFU) E[1]. Corrected samples (10 µl) were combined with equal volume of bacteria Xen 29 (~5x10^6 CFU) suspended in 10 mM sodium phosphate buffer pH 7.4, 100 mM NaCl, 1 % TSB using the precision pump of the luminometer. We measured RLU after 4 minutes of bacterial challenge.

LL-37 antimicrobial assay

Assays were performed as previously described E[1]. LL-37 (Anaspec, Fremont, CA, USA) was diluted with the peptide diluent recommended by the manufacturer. We coincubated 10 µL of LL-37 (50 µg/mL final concentration) with 10 µL of goat anti LL-37 Ab (Santa Cruz) or goat IgG anti mouse IgG (Santa Cruz) at two concentrations (5.5 and 2.75 nM). We sealed the plate with a rubber lid to avoid evaporation, and the plate was kept at 37 ºC for one hour with constant shaking (200 rpm). We then challenged the

samples with 20 µl of bacteria (~5x10^6 CFU) in an opaque white 96 well plate. RLU were registered at 4 min and percentage of live bacteria was calculated.

LL-37 neutralization in the ASL

To neutralize LL-37 in the ASL, we coincubated ASL (10 µL) with goat LL-37 Ab (10 µL) at two concentrations (7, 70 nM) or goat IgG anti-mouse IgG control (70 nM). We sealed the plate with a rubber lid to avoid evaporation, and the plate was kept at 37 ºC for one hour with constant shaking (200 rpm). Thereafter, we challenged the samples with bacteria (5x10^6 CFU) in an opaque white 96-well plate. RLU were registered at 4 min and percentage of live bacteria was calculated

Macrophage RNA microarray analysis

RNA preparation, quality analysis, and microarray analysis were done as previously described E[2]. Genome-wide macrophage mRNA expression measurements were conducted using the GeneChip Human Exon 1.0 ST Arrays (Affymetrix). We analyzed microarray data with Partek Genomics Suite, version 6.5, software (Partek, St. Louis, MO, USA). We analyzed the quality of the data and subjected to robust multi-array averaging normalization. We used paired *t*-test to compare the effect of the intervention. A p of 10^{-5} was considered significant in this analysis.

Cigarette Smoke Extract (CSE) production

CSE was made as previously described with modifications E[3]. We used a 500 µl pipette tip connected to a lit University of Kentucky Research Cigarette (Code 3R4F) in one end

and to a 50 mL syringe filled with 12.5 mL of MEM on the other end. The cigarette smoke was inhaled using the syringe and the MEM was mixed with the smoke by agitation. The resulting liquid was filtered through a 0.2 µm filter (Millipore). To ensure standardization between experiments, the absorbance was measured at 320 nm on a spectrophotometer and an optical density of 1 was defined as 100%.

PCR gene expression analysis

Airway samples were stored at -80°C in RNAlater® (Invitrogen) until we isolated RNA using the mirVana™ miRNA Isolation Kit (Invitrogen) following manufacturer's instructions. cDNA was prepared by using High Capacity cDNA Reverse Transcriptase™ kit (Applied Biosystems). A concentration of 0.0175 µg/µL of RNA in a 20 µL reaction was used to create the cDNA template. qPCR reactions were carried using Fast SYBR® Green Master Mix (Applied Biosystems) with primers (Integrated DNA Technologies) already tested in a previous publication (See supplemental Table 1) E[4]. The endogenous control was HPRT and the relative expression levels were determined using the $Fold_2$ Change based on the -ΔΔCt method (Applied Biosystems). The PCR reactions were carried out in the QuantaStudio 6 Flex following the manufacturer's instructions (Applied Biosystems).

SUPPLEMENTAL TABLE

Gene	Name	Forward	Reverse
**CAMP**	Cathelicidin Antimicrobial Peptide	ACACAGCAGTCACCAGAGCATTGT	ATTTCTCAGAGCCCAGAAGCCTGA
**CYP27B1**	1alpha-hydroxylase	AACCCTGAACAACGTAGTCTGCGA	ATGGTCAACAGCGTGGACACAAA
HPRT	housekeeping gene	GCAGACTTTGCTTTCCTTGG	AAGCAGATGGCCACAGAACT

Table 1. List of primers. From Hansdottir S et al. J Immunol 2008; 181: 7090-7099.

REFERENCES

1. Abou Alaiwa MH, Reznikov LR, Gansemer ND, et al. pH modulates the activity and synergism of the airway surface liquid antimicrobials beta-defensin-3 and LL-37. *Proceedings of the National Academy of Sciences of the United States of America* 2014;111(52):18703-8. doi: 10.1073/pnas.1422091112 [published Online First: 2014/12/17]
2. Graff JW, Powers LS, Dickson AM, et al. Cigarette Smoking Decreases Global MicroRNA Expression in Human Alveolar Macrophages. *PloS one* 2012;7(8):e44066. doi: 10.1371/journal.pone.0044066
3. Brekman A, Walters MS, Tilley AE, et al. FOXJ1 prevents cilia growth inhibition by cigarette smoke in human airway epithelium in vitro. *American journal of respiratory cell and molecular biology* 2014;51(5):688-700. doi: 10.1165/rcmb.2013-0363OC [published Online First: 2014/05/16]
4. Hansdottir S, Monick MM, Hinde SL, et al. Respiratory Epithelial Cells Convert Inactive Vitamin D to its Active Form: Potential Effects on Host Defense. *Journal of immunology (Baltimore, Md : 1950)* 2008;181(10):7090-9.
