# Supplementary material for: Effect of vitamin D3 on the antimicrobial activity of human airway surface liquid: preliminary results of a randomised placebo-controlled double-blind trial
Source: BMJ Open Respir Res. 2017 Jun 4;4(1):e000211. doi: 10.1136/bmjresp-2017-000211 (PMC5531307; doi:10.1136/bmjresp-2017-000211)
Supplement: Supplementary data [file bmjresp-2017-000211supp002.docx]

**Responses to Thorax’s reviewers**

Effect of Vitamin D3 on the Antimicrobial Activity of Human Airway Surface Liquid

C1:EDITOR Not clear what the primary comparison/s of interest are as the study design and analysis are inadequately described. I suspect the sample size and analysis methods used are inadequate as I think authors are actually interested in the interaction between intervention ( Vit D and placebo ) and smoking status, Use STROBE to help guide the essential items to report for this article?

R1: We have restructured the manuscript to follow to describe the comparison of interest (vitamin D vs placebo). We have now followed the appropriate guideline to report our results.

C2: No results are quantified eg ‘Vitamin D3 supplementation increased baseline ASL antimicrobial activity only in non-smokers’. Results for statements should include intervention effect and 95% CIs Specify study design eg ‘pre-post’ or before-after design? Not clear in abstract at all what the key comparison of interest is. P 5 there is mention of ‘placebo’ did randomization occur? Is this a sub-study from a larger study?

R2: We have now included the study design and how the randomization was done. We have also included CI for the difference in the figure legends.

C3: Population/participants? – who are these and how selected?

R3: We have now included details about the population selected.

C5: P4 – Human samples – I am not clear who the population/participants are. Inclusion/exclusion criteria, sampling approach information required.

R5: We have included the information regarding the inclusion and exclusion criteria.

C6: line 11-13 Study design/sampling of participants not clear- who receive Vit D v placebo and how was this decided.

R6: We have now included how the randomization was done.

C7: Any measure of adherence to the 90-day intervention available and examined in the analysis? If not is this considered in the discussion?

R7: We have now included information about adherence.

C8: Analysis: Statistical analysis should be improved by better describing the approach based on the comparisons of interest. So what comparisons used paired t-tests, and what used ANOVA? as these are not comparable methods: one takes into account repeat measures and the other one does not, it’s not simply a case of using ANOVA baing able to use three or more groups. They state that [paired T-test was used for comparing between interventions (I assume vit D vs placebo) but this should be an un-paired t-test. A paired t-test would be appropriate for a before and after comparison.

R8: We have expanded the statistical analysis section. We have included the test used to determine significance right next to the p-value).

C9: P7 line 14 : ‘between the interventions’ – isn’t there only one intervention? Ie Vit D?

Information on how missing or improbable data were handled?

Authors present correlations in results but not mentioned in the analysis action.

R9: We have expanded the statistical analysis section to include correlation tests used and how missing or improbable data were handled.

C10: Sample Size looks very small. Include rational for the sample size chosen based on the important comparison/s of interest. If required (i.e. more than one primary comparison of interest) please describe approach to addressing the issue of multiplicity. What level of significance are authors working to? As I suspect underpowered study.

R10: The editor has raised an important concern and we have we have restructured the manuscript accordingly. The primary comparison of interest was the effect of vitamin D on the reduction in live bacteria airway surface liquid, regardless of the smoking status. We have enough power to detect a difference between placebo and vitamin D and we have shown that vitamin D3 supplementation increases the antimicrobial activity of the ASL. We acknowledge that we might be underpowered to detect a difference in the smokers alone and we have stated this in both the result and the discussion sections. We have also included the number needed to observe a difference using the results of our study. We consider that this information might be useful in order to design better future clinical trials that include smoking as a potential confounding variable.

C11: Not sure how useful the results in table 1 as authors present results stratified by smoking status resulting in very small sample size per group and then apply large sample tests. Would it be better to summarize just by vit D v placebo so we can see how balanced these groups are? It will depend on what’s the primary comparison of interest is which I am unsure. Is it whether there a difference between Vit D and placebo adjusted for smoking status? If primary comparison between smoking and not smoking what role does the placebo comparison play? The answer to the question will best determine how to present data in table 1. If authors are trying to detect an interaction between smoking status and intervention then different analysis would be required and authors would need to use a modelling approach and I can almost guarantee this sample size would not be large enough to fit the model.

R11: We agree with the editor and restructured table 1 to reflect the editor’s opinion. We acknowledge that the sample is not large enough to model the interaction and we have restructured the manuscript to acknowledge this limitation.

C12: Bar charts should be replaced with dot-plots when sample size is this small.

R12: We have replaced most bar charts with dot plots.

Reviewer: 1

This manuscript reports results of an in vivo intervention study and a cell culture study investigating effects of vitamin D on antimicrobial responses in the lung. The authors conclude that vitamin D induces antimicrobial activity in non-smokers but not in smokers. There are some issues:

C13: The total number of participants (n=40) is modest, and the basis for the choice of sample size is not stated. Of these, only n=12 are smokers. Lack of a statistically significant effect of vitamin D sibly be due to type 2 error rather than a biological difference between groups.

R13: We have we have restructured the manuscript to respond to the concern of the sample size. Please also refer to response 9.

C14: The correct analytic approach to determine whether smoking status modifies effects of vitamin D on antimicrobial activity would be to do an interaction analysis – the P value for the interaction term (allocation*smoking status) would indicate if smoking is indeed an effect modifier. However, this analysis hasn’t been done as far as I could see.

R14: We acknowledge that the sample is not large enough to model the interaction and we have restructured the manuscript to acknowledge this limitation. Please refer to responses 9 and 10.

C15: The findings of the in vivo intervention study are further limited by the fact that the intervention did not influence 25(OH)D levels among participants. Did participants take their tablets? Did the tablets contain the advertised amount of vitamin D? In the absence of an effect of vitamin D supplementation on vitamin D status, there has to be caution about interpreting inter-arm differences in antimicrobial activity.

R15: When we combined both smokers and non-smokers, we were able to detect a significant increase in the concentration of 25(OH)D3. In addition, we have now included information about compliance.

C16: Were staff performing assays of antimicrobial activity blinded to allocation? There is potential for observation bias if not.

R16: In order to minimize observer bias, we randomized the samples onto the plate, the luminometer injected the bacteria and read RLU automatically, and the conditions were not revealed until the experiment was completed.

Minor

C17: The introduction fails to mention extra-renal CYP27B1 – this is key to the proposed mechanism of action, since CYP27B1 is expressed in respiratory epithelium and diverse leucocyte populations, and is responsible for conversion of 25(OH)D to 1,25(OH)2D in the lung.

R17: we have mentioned the extrarenal CYP27B1 in the introduction

C18: The term ‘Vitamin D3 levels’ is erroneously used where 25(OH)D levels seem to be what is meant. These are not the same thing (vitamin D3 = cholecalciferol, the parent molecule – its levels can also be measured, although this is technically challenging and these levels are not an accepted measure of vitamin D status ).

R18: We have replaced ‘Vitamin D3 levels’ with 25(OH)D3 where appropriate to avoid any confusion.

C19: Effects of vitamin D / placebo on ASL levels (Fig 4): no P values for NS comparisons are presented; I could not see mean values, standard deviations or 95% CIs for difference in means either.

R19: We have added a table with the values requested by the reviewer.

Reviewer: 2

Comments to the Author

In this study, the authors have explored the effect of vitamin D supplementation of smokers and non-smokers on the anti-staphylococcal activity of their airway surface liquid (ASL). Interestingly, this activity is increased in non-smokers, but not in smokers. The approach taken is interesting and relevant, but the description of the results raises some questions and concerns.

Major comments:

C20: Despite the fact that some participants were vitamin D (25(OH)D) deficient, vitamin D (25(OH)D3) treatment with 1000 IU/day did not increase circulating vitamin D levels. This is surprising, and raises questions regarding the reliability of the vitamin D detection assay used. Please comment.

R20: When we combined both smokers and non-smokers, we were able to detect a significant increase in the concentration of 25(OH)D3.

C21: It is not clear why the authors did not measure LL-37 levels in the ASL levels, but only provide indirect evidence for its increase.

R21: We were limited by volume and concentration of the samples to detect LL-37 by western blot. We acknowledge that this is a weakness in our study and we have stated it in both the result and discussion sections.

C22: Whereas decreased production of LL-37 in 25(OH)D3 stimulated epithelial cells from smokers may be one explanation for the lack of an increase in antibacterial activity of ASL, another explanation may be that in smokers hCAP18 was not converted to the active LL-37. Western blot analysis could have provided relevant information here.

R22: We agree with the reviewer that a different processing of hCAP18/LL-37 might affect the activity of this peptide. We included in the discussion that smokers might have a different processing of cathelicidin hCAP18/ LL-37. It has been reported that smoking increases the concentration of proteolytic enzymes such as cathepsin D and neutrophils elastase. Proteolysis can decrease LL-37 in the airways and its antimicrobial activity.

Minor comments:

C23: Introduction, page 3, last line: the authors use a general CF reference (ref 20) to indicate that impairment of AMP may be associated with disease development. Various mechanisms may indeed decrease AMP activity, including pH, salt, host and pathogen proteases, F-actin, DNA, mucus. It would seem relevant to provide a more AMP specific reference, or add this to reference 20.

R23: We have included more AMP specific references in addition to the general reference to CF.

C24: In the discussion the authors should explain the adjustment of protein concentrations of the ASL samples to 100 ug/ml. Could this introduce a bias in case vitamin D treatment affects vascular leakage (e.g. in smokers)?

R24: We have included the rationale for the adjustment in the method section. We repeated the experiment without correcting the samples and we found a similar result. In addition, we did not find a significant difference in in the protein concentration of the ASL between any of the groups (Vitamin D Vs. Placebo, Smokers Vs. Non-smokers).

C25: Is there any possible overlap between the macrophage analysis done as part of reference 21, and this study?

R25: We have included in the results a separate analysis of hCAP expression in the macrophage array to make the argument that was not different with vitamin D treatment. The main focus of the manuscript is not related to macrophages and we consider that there is no overlap.

C26: Page 7, line 4: the word “baseline” to indicate the effect of vitamin D treatment on antibacterial activity of ASL from smokers is confusing.

R26: This section has changed due to the new data analysis.

C27: Discussion, page 14, line 6-8. Please note that two studies have demonstrated an effect of vitamin D treatment on exacerbations in vitamin D deficient COPD patients in a post-hoc analysis (Lehouck et al, Ann Intern Med. 2012;156:105–14.) or in a pre-specified group (Martineau et al, Lancet Respir Med. 2014;3(2):120–30).

R27: We have deleted that paragraph to acknowledge reviewer’s comment.

C28: Figure 3. I do not think that the title matches the content of the figure. The ratio between 25(OH)D3 and 1,25(OH)2D3 (very often in the manuscript this is written as 1,25(OH)D3; please correct) is not only determined by conversion, but also by degradation. Furthermore, the authors should explain how vitamin D treatment may affect the relationship between 1,25(OH)2D3 and 25(OH)D3, without affecting the levels of these two forms of vitamin D individually (Figure 3B).

R28: We changed the title of the figure to “Vitamin D3 supplementation decreased the correlation of 25(OH)D3 to 1,25(OH)2D3” and corrected the misspelling of 1,25(OH)2D3. We have also added a paragraph explaining how vitamin D3 treatment can affect the relationship between the two forms. Increased 1,25(OH)2D3 concentrations decreases 1-α hyroxilase, which is responsible for its conversion in the kidney, and increases 24 hydroxilase, which is responsible for inactivation of both 25(OH)D3 and 1,25(OH)2D3.

C29: Figure 4 could be made clearer by including horizontal bars to indicate the mean or median.

R29: Based on C11, Bar charts should be replaced with dot-plots when sample size is this small. However, we have included the mean ± SD in the figure legend to answer to this concern.

C30: Figure 5. No information is provided on the number of individual experiments in the legend, although some information is present in the supplemental Materials and Methods. Figure 5D could be extended to also show the values with the isotype control.

R30: We have included the number of biological replicates done for every experiments in figure 5. Since we did not find a difference in the isotype control, we did not include the isotype in the experiment done in figure 5 D.

C31: Supplemental materials and methods, page 2. First paragraph, last line. Please add concentration of the sodium phosphate buffer (10 mM).

R31: We have added the concentration of sodium phosphate buffer.

C32: Supplemental materials and methods, page 3. The authors now refer to a mouse anti-LL-37, whereas in the main manuscript they mention a goat antibody (page 8).

R32: We have corrected this mistake. In both sections it now says goat anti LL-37 and goat IgG anti mouse IgG.

C33: On the same page (page 3), the word “immunoprecipitation” is incorrect. In fact the antibody neutralizes the LL-37 activity, but no evidence of immunoprecipitation is shown.

R33: We agree with the reviewer and we have substituted the word “immunoprecipitation” with the word “neutralization”.

C34: Supplemental materials and methods, page 4. I assume the Millipore filter was 0.2 um and not nm?

R34: We have corrected this mistake.

C35: Supplemental materials and methods, page 5. The description of the experiments with human airway epithelial cells is not clear. Did they treat the cells for 4 days with vitamin D, and next in the last 24 hours with CSE?

R35: We have changed the wording to clarify what we did. First, we exposed overnight to 25(OH)D3 (n=8), or 1,25(OH)2D3 (n=5) in the presence of CSE or control and analyzed the gene expression of CAMP. In separate experiment, we exposed cells to only 25(OH)D3, 1,25(OH)2D3, CSE, or control for four days and analyzed the relative gene expression, of CAMP and CYP27B1 separately (n=4).
