# Supplementary material for: Effect of vitamin D3 on the antimicrobial activity of human airway surface liquid: preliminary results of a randomised placebo-controlled double-blind trial
Source: BMJ Open Respir Res. 2017 Jun 4;4(1):e000211. doi: 10.1136/bmjresp-2017-000211 (PMC5531307; doi:10.1136/bmjresp-2017-000211)
Supplement: Supplementary data [file bmjresp-2017-000211supp003.docx]

- Title Identification of the study as randomised
- Authors* Contact details for the corresponding author
- Trial design Description of the trial design (eg, parallel, cluster,non-inferiority)

Methods

- Participants Eligibility criteria for participants and the settings
- where the data were collected
- Interventions Interventions intended for each group
- Objective Specifi c objective or hypothesis
- Outcome Clearly defi ned primary outcome for this report
- Randomisation How participants were allocated to interventions
- Blinding (masking)
- Whether or not participants, care givers, and those assessing the outcomes were blinded to group assignment
- Results
- Numbers randomised
- Number of participants randomised to each group
- Recruitment Trial status
- Numbers analysed
- Number of participants analysed in each group
- Outcome For the primary outcome, a result for each group and the estimated effect size and its precision
- Harms Important adverse events or side-effects
- Conclusions General interpretation of the results
- Trial registration Registration number and name of trial register
- Funding Source of funding
